# Circadian regulation of extracellular vesicle biogenesis, composition, and release

**DOI:** 10.1038/s44323-025-00053-1

**Published:** 2025-11-03

**Authors:** Jonathan D. Church, Elizaveta Kadukhina, Ignacio Aiello, Carla V. Finkielstein

**Affiliations:** 1 Fralin Biomedical Research Institute at VTC, Virginia Tech, Roanoke, VA USA; 2 Translational Biology, Medicine, and Health, Fralin Biomedical Research Institute at VTC, Virginia Tech, Roanoke, VA USA; 3 Department of Biological Sciences, Virginia Tech, Blacksburg, VA USA; 4 Molecular Diagnostics Laboratory, Fralin Biomedical Research Institute at VTC, Virginia Tech, Roanoke, VA USA

**Keywords:** Circadian rhythms, Circadian rhythm signalling peptides and proteins

## Abstract

Circadian rhythms in mammals are governed by cell-autonomous oscillators that synchronize physiological functions through central and peripheral communication. Extracellular vesicles (EVs) mediate intercellular and systemic signaling, with time-of-day-dependent release and cargo composition regulated by the circadian clock. This regulation involves both transcriptional and post-transcriptional mechanisms affecting genes involved in EV biogenesis. Studies in diverse models show EVs impact physiological and pathological processes, including inflammation, cardiovascular disease, chronic kidney disease, and cancer. EVs also serve as important biomarkers, especially in cancer. The accompanying article explores how clock proteins regulate multiple aspects of EV biology and highlights the significance of circadian dysregulation in EV-associated pathologies. Understanding EV-mediated signaling within the framework of circadian rhythms is critical for improving disease diagnosis, informing treatment strategies, developing therapeutic applications, and enhancing the diagnostic utility of EVs. This insight may pave the way for novel chronotherapy approaches in clinical practice.

## Introduction

Extracellular vesicles (hereafter EVs) are small, lipid-bilayer-enclosed particles released by cells and incapable of self-replication^1^. This definition includes both naturally occurring and engineered EVs, albeit the later will not be discussed in this article. Originally thought to be a method of waste disposal, EVs are now recognized as key players in various physiological processes, including cell-cell communication, autophagy, membrane protein recycling, immunomodulation, and cellular homeostasis^2^.

EV release is cell-type-dependent, varying in both particle number and composition, and its heterogeneity influences the progression of various pathological conditions^2,3^. For example, cancer cells release an abundance of EVs with altered composition that promote angiogenesis and metastasis^4–6^. Environmental factors such as hypoxia and low pH, which are characteristic of the tumor microenvironment, further modulate EV release and composition^3,7–9^. Given their significance in both normal and pathological processes, understanding the mechanisms underlying EV biology is of major interest. Although much progress has been made, many aspects of their biology remain to be elucidated, particularly regarding their spatial-temporal regulation and the physiological and environmental factors that modulate these processes. In this context, the circadian clock, an endogenous and autonomous biological mechanism that generates and maintains physiological and behavioral changes following a ~24-h cycle, has emerged as a promising area of research for regulating EV biogenesis and release.

In 2014, and more recently in 2023, an interdisciplinary group of scientists from the International Society of Extracellular Vesicles published a position article on the latest developments in basic EV research, titled “Minimal Information for Studies on Extracellular Vesicles,” or MISEV 2023^1^. In addition to summarizing state-of-the-art approaches for purifying, characterizing, and studying EVs, the authors encouraged researchers to record the time of day of the EV collection to account for circadian variation^1^. Biomarker research has gradually adopted this guideline, with more recent publications specifying the time of day at which samples were collected. As a result, our review summarizes the current state of EV research, particularly focusing on how the biochemical and cellular processes involved in their biogenesis and release are modulated by the circadian clock. Furthermore, by analyzing multiple genome-scale time-datasets, we identified rhythmic transcripts that are relevant to EV biology across various tissues, with an emphasis on how these oscillations may influence EV function and their potential applications as time-sensitive biomarkers.

The circadian clock operates in a quasi-hierarchical system, with a central oscillator located in the suprachiasmatic nuclei (SCN) of the anterior hypothalamus. This central pacemaker receives photic signals from intrinsically photosensitive melanopsin retinal ganglion cells (ipRGCs) via the retinohypothalamic tract and synchronizes circadian clocks throughout the brain and peripheral tissues. It controls the phase of various peripheral oscillators through direct neuronal projections, the autonomic nervous system (ANS), and neuroendocrine signals, regulating the rhythms of secondary oscillators (e.g., pineal gland, pituitary gland, adrenal glands) to adapt to environmental changes^10,11^. The oscillations of peripheral tissue explants and cell cultures rapidly dampen and desynchronize, highlighting the critical role of the SCN in maintaining the robust rhythmicity of peripheral clocks. In support of this model, circadian clocks have been identified in virtually every tissue, organ, and isolated cell^12,13^. Glucocorticoids (GCs) are a well-established example of systemic circadian coupling regulated by the suprachiasmatic nucleus (SCN). In response to light cues, the SCN stimulates the hypothalamus to release corticotropin-releasing hormone (CRH), which in turn triggers the pituitary gland to secrete adrenocorticotropic hormone (ACTH). ACTH then acts on the adrenal cortex to promote the rhythmic release of GCs, which synchronize peripheral clocks by modulating clock gene expression. Dexamethasone, a synthetic GC steroid, is routinely used to synchronize the circadian clock in vitro and in vivo by acting on glucocorticoid response elements (GREs) and rapidly inducing *PER1* expression^14,15^. However, studies of SCN-lesioned mice challenge this model by demonstrating the ability of peripheral tissues to generate self-sustaining rhythms in gene expression that persist independently of the SCN. This has led to the idea that the SCN is necessary for synchronizing the phase of independent, self-sustaining clocks, with circadian coupling necessary to coordinate physiological processes and adapt to changing environmental conditions^16–18^.

Coupling occurs across different levels, including within an individual cell (e.g., molecular coupling), locally between neighboring cells (e.g., intercellular coupling), and distally between peripheral tissues^19^. Intracellular and systemic coupling between non-SCN tissues is achieved through gap junction signaling, paracrine signaling, and various humoral signals (e.g., hormones, metabolites, and other blood-borne signals)^11,19^. Cytoplasmic concentrations of Ca^2+^/cAMP and the resulting downstream signaling are known to be critical for pacemaking in organotypic SCN brain slices^20,21^. Outside of the SCN, the concentrations of K^+^ and Ca^2+^ ions also influence circadian oscillator properties by altering membrane potential^22^. It is, therefore, thought that individual cells can be coupled by cytosolic ion concentrations and the transfer of intracellular ions or secondary messengers through gap junctions^19,21^. Conditioned media (CM) from cells is another source of secreted factors that serve to couple the circadian clock of individual cells in a paracrine manner. CM from human and murine cells has been found to induce phase delays in U2OS reporter cells via TGF-β/SMAD4 signaling and downstream CREB response elements (CRE) activation of *PER2* expression^23^. Different coupling mechanisms of the circadian clock enable efficient communication of circadian information across various spatial and temporal scales. Time-sensitive feedback across organizational levels necessitates diverse coupling mechanisms, suggesting that additional, yet-to-be-identified mechanisms likely exist, contributing to the robustness of the circadian system.

More recently, there has been a conceptual shift in our understanding of EVs, positioning them as potential systemic synchronizing agents capable of coordinating cellular functions across various tissues. Accordingly, we propose that EVs are ideal candidates for circadian coupling due to their: (i) relative abundance and stability in circulation, (ii) high degree of regulation, and (iii) ability to transport bioactive cargo (e.g., proteins, metabolites, RNA, and miRNAs) over long distances. This new perspective on paracrine signals for systemic communication in the circadian field highlights the relevance of EVs and offers a transformative view of how communication and synchronization might be achieved.

### Molecular mechanisms driving the mammalian circadian clock

**Fig. 1:**
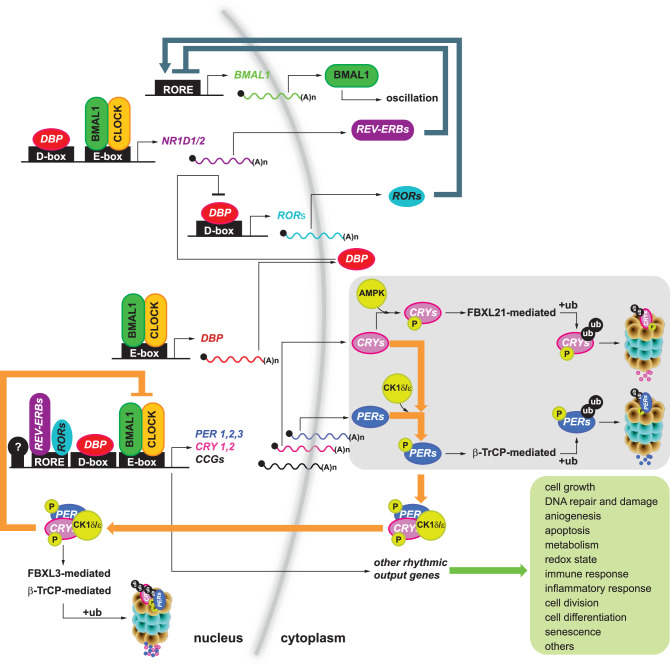
Molecular mechanism of the mammalian circadian clock. The schematic illustrates the interplay among clock components that regulate circadian oscillations. CLOCK, or its paralogue NPAS2, heterodimerizes with BMAL1 and binds to regulatory E-boxes in *PER1-3*, *CRY1-2*, and other clock-controlled genes (*CCGs*), driving their expression. PER and CRY proteins accumulate in the cytoplasm, undergo post-translational modifications by CKIδ/ε, and are translocated to the nucleus. In the nucleus, the PER:CRY:CKIδ/ε complex inhibits the transcriptional activity of CLOCK:BMAL1, repressing *PER* and *CRY* expression (orange arrow circuit). In a secondary loop, CLOCK:BMAL1 activates the transcription of *NR1D1* and *NR1D2*, whose protein products (REV-ERBs) bind to RORE elements, competing with *RORα, β*, and *γ* to regulate BMAL1 expression (dark blue arrow circuit). An additional regulatory loop involves CLOCK:BMAL1-driven expression of DBP, which binds to D-box elements in core clock components, modulating *ROR* and *PER* transcription. The phosphorylation of PER2 and its ubiquitin-mediated degradation are fine-tuned by additional kinases (e.g., CK2) and ubiquitin ligases, such as β-TRCP. The PERs’ binding partners, CRY proteins, are targeted by the cellular stress sensor kinase AMPK, and their degradation involves the E3-ubiquitin ligases FBXL3 and FBXL21. Lastly, several cellular pathways regulated by circadian clock components are listed in the green box. For simplicity, the following gene acronyms are used: CLOCK, NPAS2, BMAL1, PER1-3, CRY1-2, CKIδ/ε, NR1D1-2, REV-ERBs, RORE, RORα/β/γ, CK2, β-TRCP, AMPK, FBXL3, and FBXL21. Their full descriptions are provided in the main text. Additional abbreviations include: ub ubiquitin, (A)n poly(A) tail, P phosphorylation, and “?” refers to additional transcriptional regulators.

The core molecular clock operates through interlocked transcriptional-translational feedback loops (TTFLs) driven by processes that include protein synthesis and degradation, compartmentalization, and post-translational modification (Fig. 1). In addition, clock proteins regulate the rhythmic expression of clock-controlled genes (*CCGs*), which are responsible for regulating various physiological processes, such as metabolism, immune response, and cellular repair, to name a few^24,25^. Over the last several decades, much work has been done to characterize the various transcriptional, post-transcriptional, and post-translational processes regulating the circadian clock in different organisms [^24,26–28^ and references within]. Briefly, in mammals, the basic helix-loop-helix (bHLH) Per-ARNT-Sim (PAS) circadian locomotor output cycles kaput (CLOCK) transcription factor, or its paralogue neuronal PAS domain protein 2 (NPAS2), heterodimerize with brain and muscle ARNT-like 1 (BMAL1) and bind regulatory E-boxes in core clock genes *period* (*PER1, 2*, and *3*) and *cryptochrome* (*CRY1, 2*) as well as other output *CCGs*, driving their expression. PER and CRY proteins accumulate in the cytosol, are post-translationally modified by casein kinase 1δ and ε (CKIδ/ε), and translocate to the nucleus where PER:CRY:CKI(δ/ε) complex negatively regulates the transcriptional activity of CLOCK:BMAL1, resulting in the repression of *PER* and *CRY* expression (Fig. 1). In a second regulatory loop, CLOCK:BMAL1 also drives the transcription of the *nuclear receptor subfamily 1 group D member 1 and 2* (*NR1D1* and *NR1D2*), whose protein products (REV-ERBs) bind to retinoic acid-related orphan receptor-binding elements (ROREs), competing with ROR-related orphan receptor (*RORα, β*, and *γ*), thus balancing the daily levels of BMAL1 expression (Fig. 1). An additional regulatory loop is driven by CLOCK:BMAL1-mediated expression of the acidic amino acid-rich basic leucine zipper (PAR bZIP) transcription factor [D-box binding PAR bZIP transcription factor (DBP), thyrotroph embryonic factor (TEF), hepatic leukemia factor (HLF)] genes, which bind to D-box elements of core clock components to regulate *ROR* and *PER* transcription [Fig. 1 ^24,29^].

Post-translational modifications, particularly phosphorylation, as well as SUMOylation, ubiquitination, acetylation, O-GlcNAcylation, and ADP-ribosylation, are also relevant in maintaining the pace of the clock, as they have been identified in mechanisms across kingdoms^26^. Casein kinase 1δ/ε (CK1δ/ε) phosphorylates PER2 either at unprimed sites, without prior phosphorylation, or more efficiently at primed sites where adjacent residues are already phosphorylated. This differential targeting supports a proposed “phosphoswitch” mechanism that modulates PER2 stability and adjusts the pace of the circadian clock, allowing it to compensate for factors such as temperature changes [for review see^30^]. The interplay between phosphorylation of PER2 and ubiquitin-mediated degradation is fine-tuned and also mediated by additional kinases [i.e., CK2^31,32^] and the F-box/WD repeat-containing protein 1 A (β-TRCP)^33,34^, albeit phosphorylation-independent mechanisms of PER2 degradation exist via RING-finger E3-ubiquitin ligases^35^ (Fig. 1). PER proteins’ binding partners, the CRYs, are phosphorylated by the cellular stress sensor AMP-activated protein kinase (AMPK). Unlike CK1δ/ε, which sequentially phosphorylates adjacent serine/threonine residues following an initial phosphorylation event (a process known as tandem phosphorylation), AMPK targets specific phosphorylation sites without promoting further phosphorylation nearby. Degradation of CRYs subsequently requires the F-box E3 ubiquitin ligases FBXL3 and FBXL21^36^ (Fig. 1). Lastly, components of the auxiliary loops, such as REV-ERBα, have been reported as targets of glycogen synthase kinase 3 (GSK3) and cyclin-dependent kinase 1 (CDK1). The former prevents REV-ERBα-mediated degradation by the E3-ligase HUWEI, whereas the latter facilitates FBXW7-mediated degradation and influences circadian oscillation^37,38^. Other modifications, such acetylation and O-GlucNAcylation in PER2, which arise from the cell’s metabolic state, also influence the extent of CKIδ/ε-mediated phosphorylation as well as the clock’s output^39–41^.

The stability of CLOCK and BMAL1 is also regulated post-translationally. Phosphorylation of BMAL1 at specific residues differentially regulates its localization, stability, and activity. Phosphorylation at serine 90 (by CK2α) promotes nuclear localization^42^, while phosphorylation at Ser42 (by S6K1) enhances BMAL1-dependent translation^43^, and phosphorylation at the same site (by AKT2) inhibits its nuclear entry^44^. In addition, phosphorylation at Ser17 and Thr21 (by GSK3β) promotes BMAL1 degradation through ubiquitination^45^. These site-specific modifications are recognized by E3 ligases that target phosphorylated substrates conjugated with SUMO or ubiquitin^46,47^. Furthermore, acetylation also modulates BMAL1’s transcriptional activity^48^. Phosphorylation of CLOCK is a complex process, involving multiple sites targeted by various kinases. This modification appears to modulate transactivation and localization; however, its broader role in clock regulation remains incompletely understood [for review see in ref. ^49^].

The circadian clock is tightly regulated post-transcriptionally, as shown in fruit fly and mouse models, with rhythmic mRNA accumulation and translation driven by processes like alternative splicing, mRNA modifications, RNA-binding proteins, and miRNA activity [for review see in ref. ^27^ and references within]. Notably, poly(A) tail length influences the stability and rhythmic expression of genes, with shorter tails generally associated with enhanced translational efficiency^50^. Rhythmic regulation of poly(A) tail length has been linked to the circadian control of deadenylation pathways. Among these enzymes, nocturnin was originally identified as a rhythmic deadenylase implicated in modulating poly(A) tail length in tissues with robust circadian activity^51,52^. However, more recent findings suggest that nocturnin may instead function as a NADPH phosphatase^53^, prompting a reassessment of its mechanistic role in circadian RNA regulation. Lastly, microRNA (miRNA)-mediated post-transcriptional regulation of core clock transcripts has garnered significant attention, as both in silico and experimental studies have predicted that several miRNAs may target core clock genes for degradation in *Drosophila*, mice, and human cell lines. These targets include *clk*, *tim*, and clockwork orange (*cwo*) in *Drosophila*; *Per1*, *Per2*, and *Per3* in mice; and *BMAL1*, *CLOCK*, *PER*, and *CRY1* in human lines. All these mRNA transcripts often exhibit multiple miRNA base-pairing sites in their 3’UTR, which may influence gene expression, and thereby sustaining the pace of the clock^54–61^. However, the functional relevance of miRNAs in core clock regulation remains under active investigation. For example, in Du et al.^62^, liver-specific knockout of *Dicer* in mice maintained under 12:12 LD conditions and with an intact SCN did not disrupt liver clock oscillations, although elevated expression of certain clock genes (*Per1*, *Per2*, *Cry2*) was observed^62^. These findings suggest possible miRNA involvement in fine-tuning transcript levels, even if core rhythmicity is maintained. Moreover, free-running liver explant data from the same study showed a non-significant trend toward period lengthening, possibly indicative of a masking effect. In contrast, a study by Chen et al.^63^ reported significant period shortening in global *Dicer* knockout models, further highlighting the complexity and context-dependence of miRNA contributions to circadian regulation^63^. Together, these findings suggest that while miRNAs may not be essential for generating core circadian rhythms, they likely play a modulatory role in fine-tuning clock gene expression and maintaining circadian robustness under specific physiological conditions.

While the mechanisms listed above are the primary drivers of the circadian clock, additional factors and interactions add to the complexity and precision of circadian regulation in mammals. The clock’s transcriptional activity is further regulated by rhythmic DNA polymerase activity and the overall chromatin landscape, with post-translational histone modifications (e.g., acetylation, methylation, and ubiquitylation) influencing the core heterodimer complex’s access to promoter regions in clock-controlled genes^24,26^.

### Biology of extracellular vesicles (EVs) in physiological and pathological scenarios

**Fig. 2:**
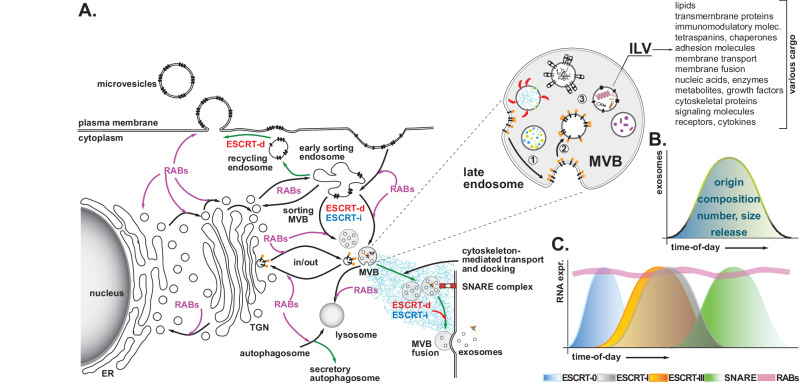
Mechanisms of EV sorting and their circadian dynamics. **A** Schematic representation of most well-defined intracellular routes involved in the generation, loading, and release of microvesicles and exosomes. The cartoon depicts the initial steps of the process, where inward budding of the plasma membrane leads to the formation of early sorting endosomes, which then fuse and mature into late endosomes - of which the multivesicular body (MVB) is an example. Cargoes targeting the multivesicular structure originate from the endocytic pathway or various intracellular routes, including the trans-Golgi network (TGN), endoplasmic reticulum (ER), recycling pathways, and retrograde transport, among others. Intraluminal vesicles (ILV), generated by inward budding of the endosomal membrane and subsequent release into the lumen (inset, steps 1 to 3), contain cargoes of various molecular nature, including lipids, proteins, nucleic acids, and even multimeric complexes. Exosome accumulation results from the docking and fusion of MVBs with the plasma membrane, followed by ILV release. This tightly regulated process involves cytoskeleton reorganization, various members of the RAS-associated binding protein (RAB) family of small GTPases, and the SNARE complex. Green lines indicate secretory pathways. ESCRT-d and ESCRT-i refer to ESCRT-dependent and ESCRT-independent pathways, respectively, highlighting distinct mechanisms of extracellular vesicle formation and cargo sorting. **B** Two-dimensional plot illustrating various aspects of exosome biology, including origin, composition, and release, all under circadian control. **C** Two-dimensional plot summarizing the temporal distribution of components involved in exosome biosynthesis and release, categorized into five key regulatory groups [i.e., ESCRT-0 (blue), ESCRT-I (gray), ESCRT-III (orange), SNARE (green), RABs (purple)] based on their circadian phase. The data are derived from mouse transcriptome information summarized in Table I.

There are two major classes of lipid-enclosed EVs, which are primarily defined by their cellular origin: exosomes (from internal compartments) or microvesicles/ectosome (by outward budding and fission of the plasma membrane) (Fig. 2A)^1^. Exosomes are small, typically less than 150 nm in diameter, and originate from the multivesicular body (MVB), an intermediate endosomal compartment within the endolysosomal pathway. In contrast, microvesicles (hereafter MVs) range in size from 100 nm to 1 μm on average. However, it is recommended to avoid using specific size cut-offs to define the different populations of EVs, as overlapping sizes exist^64–66^. Various other subclasses of EVs have been described in the literature and are categorized by morphology, cargo content, and cell type from which originate (e.g., oncosomes, apoptotic bodies, and arrestin-mediated microvesicles), a full discussion of which can be found elsewhere^6^.

EVs are highly heterogeneous in size, composition, and biochemical markers, making the isolation and characterization of specific EV subtypes challenging using current methods. This heterogeneity poses challenges in studying the role of EVs in normal and pathological processes, as virtually every cell type releases EVs, with multiple populations differing in quantity and phenotype. Single-cell studies expose this variability. Using a microchip platform to profile single-cell EV secretion, Ji et al. demonstrated that oral squamous cell carcinoma (OSCC) cells preferentially released EVs with distinct secretion profiles, enabling the clustering of different functional subgroups with unique invasive features^67^. Interestingly, the authors also identified outlier subgroups of cells in which EV secretion was either abrogated or exacerbated (named “super EV secretors”)^67^.

Pioneering work established that EVs, specifically exosomes, could transport functional mRNAs and miRNAs from one cell to another^68^. Subsequent deep-sequencing of small RNAs released by immune cells showed an enrichment in sequences targeting structural, regulatory, and coding regions, suggesting a selective process for loading nucleic acids into vesicles to exert a functional control of biological processes in target cells^69^. Although the mechanisms that sort nucleic acids into exosomes remain largely elusive, many groups have pointed to the existence of target motifs and the formation of RNA-protein binding complexes within membrane microdomains that precede sorting^70,71^. Today, the characterization of molecules loaded into EVs has expanded to include transport proteins, RNAs (e.g., mRNAs, microRNAs, circRNAs, non-coding RNAs), DNA (genomic DNA, mitochondrial DNA, single strand DNA, and tumor DNA), growth factors, cytokines, enzymes, metabolites, and other bioactive molecules^72^. Importantly, the composition of EVs varies by cell type, activation state (e.g., immune cells), environmental factors, and potentially time of day, a topic discussed in more detail in a later section, reflecting the diverse functions of EVs under different physiological conditions.

Since the discovery of EVs containing double-stranded DNA reflecting the mutational status of cancer cells, tumor-derived EVs have attracted interest as potential screening, diagnostic, and prognostic biomarkers in cancer^73,74^. Circulating EVs are ideal candidates due to their stability in circulation, presence in various biofluids (e.g., blood, lymph, saliva, milk, semen, cerebrospinal fluid), and distinct molecular profiles (e.g., surface proteins, miRNAs, proteins), which can be linked to their cellular origin, enabling specific diagnoses.

EVs from different cancers and stages are known to be selectively enriched with specific molecular loads. For example, mutations in the Kirsten rat sarcoma virus (*KRAS*) gene—a well-known driver of pancreatic ductal adenocarcinoma (PDAC)—and the tumor suppressor *TP5*3 have been identified in nucleic acids from PDAC-derived EVs obtained through liquid biopsies^75^. In a meta-analysis of 39 studies on pancreatic cancer, including more than 2000 patients, circulating EVs were shown to be selectively enriched with miRNAs (e.g., miR-21, miR-10b, miR-451a, miR-106b, miR-155, miR-181a, miR-191, miR-1246, and miR-20a) and proteins [e.g., glypican-1 (GPC-1), ephrin type A receptor 2 (EphA2)] in patients compared to healthy individuals and those with benign pancreatic diseases. Furthermore, circulating EVs have shown high diagnostic sensitivity and specificity in distinguishing early-stage pancreatic cancer (stage I and II) from healthy individuals. In this context, sensitivity refers to the ability to correctly identify those with the disease (true positives), while specificity refers to the ability to correctly identify those without the disease (true negatives). This strong performance highlights the potential of circulating EVs as valuable biomarkers for early pancreatic cancer detection^80^. Presently, EVs are being investigated as potential diagnostic biomarkers for various cancers, including endometrial, bladder, colorectal, lung, glioblastoma, and hepatocellular carcinoma^71,76–83^.

Despite recent advances in the development of EV-based biomarkers, their clinical implementation remains hindered by the lack of standardized methods for normalizing EV isolation from different biofluids and heterogeneous patient populations. In this context, a deeper understanding of the impact of time-of-day sample collection and the role of circadian rhythms in EV biogenesis and release will be critical in advancing the use of EVs as reliable biomarkers and therapeutic tools.

### Multivesicular body biogenesis and cargo sorting

Exosome biogenesis is a highly regulated process involving multiple steps and intermediary structures, as extensively reviewed by^64,66^. In brief, the process begins with the endocytic pathway and the formation of “early endosomes,” which arise from the inward budding of the plasma membrane (Fig. 2A). Early endosomes then fuse and mature into late endosomes, structures responsible for sorting cargo either to the plasma membrane for recycling or to the lysosomes for degradation. A subtype of late endosomes, the multivesicular body (MVB), is characterized by the presence of small intraluminal vesicles (ILVs), which form by the invagination of the late endosomal membrane and contain various types of cargo (e.g., proteins, nucleic acids). The MVBs also dynamically interact with other organelles, such as the trans-Golgi network and lysosomes, to regulate MVB’s fate and vesicle composition. In the final step, the MVB fuses with the plasma membrane, releasing ILVs, now termed exosomes, into the extracellular milieu (Fig. 2A).

The biogenesis and loading of ILVs occur within the lumen of endosomes, involving both canonical and non-canonical sorting mechanisms. In this process, cargo accumulates on microdomains of the endosomal membrane before inward budding takes place^84,85^. The canonical endosomal sorting complex required for transport (ESCRT)-dependent pathway requires the stepwise recruitment of five complexes (ESCRT-0, I, II, III, and the ESCRT accessory protein AAA-ATPase VPS4), each playing distinct roles in ILV formation, including sorting of ubiquitinated protein cargo, membrane deformation, and vesicle scission (Fig. 2A).

The ESCRT-0, I, and II complexes primarily function in the recruitment and sorting of ubiquitinated cargo to the limiting endosomal membrane^86^. Along with lipid-driven mechanisms that are not yet fully understood, they facilitate membrane invagination. The ESCRT-0 complex consists of two subunits: hepatocyte growth factor-regulated tyrosine kinase substrate (HRS) and signal transducing adaptor molecule 1 (STAM1). The HRS subunit binds to phosphatidylinositol 3-phosphate (PtdIns3P) *via* its FYVE domain, directing trafficking to the endosomal membrane. Both HRS and STAM1 contain ubiquitin-binding domains, aiding in sorting ubiquitinated cargo into developing vesicles. The ESCRT-I complex, composed of four protein subunits - vacuolar protein sorting 23 [VPS23, also known as TSG101], VPS28, VPS37A, and multivesicular body subunit 12 (MVB12), which works with ubiquitin-associated protein 1 (UBAP1), may allow ESCRT-III recruitment independent of ESCRT-II. Following ESCRT-0 localization, ESCRT-I is recruited *via* interaction between HRS and TSG101. The ESCRT-II complex is then recruited through interaction between VPS28 and the RNA polymerase II elongation factor (ELL)-associated subunit. The ESCRT-III and VPS4 ATPase complexes facilitate vesicle scission of the MVB lumen and recycle ESCRT subunits, completing ILV assembly^87^. The importance of ESCRT and VPS4 in various aspects of ILV biogenesis and exosome secretion was highlighted in a landmark study using a FACS-based assay to quantify exosomes secreted from cells in which individual genes were knocked down using targeted small hairpin RNAs^88^.

Other variations in membrane budding and cargo clustering exist (Fig. 2A). For instance, the syndecan-syntenin-ALG-2 interacting protein X (ALIX) operates independently of the early ESCRT components but still requires VPS4 for the scission step^89^. This mechanism is particularly relevant in signaling events where heparan sulphate on the cell membrane is essential for cargo engagement and docking (e.g., some receptor-ligand interactions) as well as in budding processes (e.g., Epstein-Barr virus release)^90^. Lastly, ESCRT-independent mechanisms of exosome biogenesis and uptake represent an emerging area of research. Evidence supports the formation of ceramide, because the enzymatic cleavage of sphingomyelin by neutral sphingomyelinase type 2 enzyme (SMase2), and its accumulation in lipid raft microdomains, act as catalytic events in ILV and exosome biogenesis^91^. Cargo sorted by this pathway includes lipids (e.g., cholesterol) and integral membrane proteins such as tetraspanins (e.g., CD63, CD81), proteoglycans, and flotillins (e.g., FLOT-1 and FLOT-2). However, the contribution of these various cargos to exosome biogenesis is conflicting and depends on cell type and physiological or pathological stimuli^92,93^.

### Diurnal variation in extracellular vesicle release, size, and composition in homeostatic and disease conditions

The presence of secreted membrane vesicles, broadly defined, dates to the late 1960s when Wolf, in an attempt to better characterize a “clotting activity” originating from platelet-free plasma, identified particulate coagulate material that he referred to as “platelet-dust”^94^. This low-density platelet-dust was described as lipid-rich with coagulative properties, and distinct from intact platelets, red blood cells, or chylomicra^94^. In a series of images, Wolf showed that platelet-dust isolated from serum was morphologically identical to those extruded from the cytoplasm of ruptured platelets, with a diameter of ~500 Å (50 nm), occasionally clustering to form clumps of up to 1 mm^94^. Particles containing procoagulant activities were later isolated from other immune cells and, based on their biogenesis, defined as microvesicles [e.g.^95^, and^96^].

As early as the 1980s, researchers identified time-of-day variations in the volume, surface density, number, and transport activity of microvesicles^97,98^ (Fig. 2B). The study examined the ultrastructure of rat gastric parietal cells at six timepoints over 24 h under two feeding conditions: prolonged fasting (40 h) and mid-light restricted feeding (4 h)^97^. Rats were maintained on a 12:12 light-dark cycle, providing a circadian cue. Measurements included the surface density (Sv) of microvesicles, extracellular vesicle (EV)–like structures, among other parameters. The study showed circadian variation in microvesicular density, suggesting a rhythm in gastric EV-related activity that persisted even during fasting^97^. This rhythm peaked during the late dark phase, aligning with previous findings in ad libitum-fed rats. Notably, restricted feeding during the rest phase induced a ~180° phase shift in this rhythm, indicating that feeding time can act as a dominant circadian cue for EV-related processes in the stomach. These findings support the idea that both light-dark cycles and feeding schedules modulate EV dynamics in gastric tissue^97^. In a separate series of experiments, Bourdel et al. demonstrated circadian variations in sodium-gradient stimulated transport of L-proline in rat liver microvesicles collected at different times^98^. While these studies provided some of the first evidence that circadian rhythms influence vesicular content variation, they were limited by the absence of standardized methods for isolating and characterizing different subclasses of vesicles.

Fast forward to today, there is now accumulating evidence suggesting that circadian rhythms regulate diurnal variations in the release of diverse EVs (Fig. 2B). In a study investigating the increased risk of thrombotic events occurring in the late-morning, Madden et al. analyzed the distribution of pro-coagulant microparticles, specifically vascular cell adhesion molecule-1 (VCAM-1) and tissue factor (TF)-associated microparticles, in blood samples from healthy individuals collected over a 24-h period^99^. The findings revealed a significant circadian variation in the number of VCAM-1 positive microparticles in circulation, with a similar trend, albeit not significant, observed for TF microparticles^99^. While it is not possible to determine whether the diurnal variation reported in this study reflects changes in overall microparticle release or a shift in microparticle composition, as the absolute number of microparticles in circulation was not reported, the results still underscore the influence of circadian rhythms on microparticle dynamics.

Given the importance of EVs in blood as disease biomarkers, an accurate assessment of circadian variation in particle size and number is warranted. In an initial attempt to address this, Danielson et al. used nanoscale flow cytometry (nanoFCM) to examine the circadian variation of EVs in blood samples collected from six healthy adults at three time points over a 24-h period^100^. The authors showed that the relative quantitative sizing and counting of EVs in plasma varies throughout the day. While inter-subject, time-of-day-dependent variations in both EV parameters were not entirely consistent, every subject exhibited some level of circadian variation, with EVs being, on average, larger at later times in the day^100^. As the authors point, while nanoFCM effectively detects vesicles as small as 100 nm, further methodological refinement and larger studies, where participants are subjected to standardized conditions (e.g., food intake, exercise), are needed to enable accurate quantification and to validate EVs as time-sensitive biomarkers.

The circadian oscillation of microvesicles has been studied in pathological contexts, such as the patho-mechanism of obstructive sleep apnea (OSA), and as potential disease biomarker^101^. Initial studies in this area were technically sound but largely inconsistent, likely due to differences in the time of day when samples were collected. To address this, Bikov et al. analyzed changes in circulating levels of microvesicles in patients with OSA, healthy individuals, and those treated with continuous positive airway pressure (CPAP) therapy^101^. In this study, a significantly higher variation in the number of CD41^+^ and Annexin V^+^ microvesicles was detected in OSA patients, with a peak at 5 P.M., compared to healthy controls^101^. Furthermore, a significant correlation was found between the presence of CD41^+^ microvesicles and several indices of OSA severity, including the apnea-hypopnea index, respiratory disturbance index, oxygen desaturation index, the percentage of total sleep time spent with SpO_2_ < 90%, minimum saturation, and average oxygen saturation. Interestingly, the diurnal variation of CD41^+^ and Annexin V^+^ microvesicles was blunted after two months of CPAP treatment^101^. In another study, Koritzinsky et al. quantified the secretion of small EVs (less than 200 nm) in urine samples from a rat model of kidney ischemia-reperfusion injury using nanoparticle tracking analysis and tumor susceptibility gene 101 (TSG101) marker detection^102^. The animals exhibited a variation in the concentration of small EVs excreted in urine over a 24-h period, peaking during the dark phase when rats are more active^102^. Notably, this rhythmic pattern of small EV excretion in urine was found to be independent of sex, fed-fasting state, hydration state, and acute kidney injury^102^. Similarly, Bazie et al. analyzed blood samples from patients chronically infected with HIV undergoing antiretroviral therapy, as well as uninfected individuals, to assess the number, size, and miRNA content of EVs at two time points during the day (10 A.M. and 10 P.M.)^103^. The results showed that the number and size of EVs were significantly larger in plasma collected in the morning compared to the evening in both groups. However, miRNA abundance, specifically the expression of five distinct miRNAs, differed only in the control group and was not restored in HIV-infected individuals, even when receiving viral-suppressive therapy^103^. It is important to note that these findings are based on data from only two time-points, so, despite their significance, they should be interpreted as daily variations until proper circadian studies are conducted.

In addition to time-of-day-dependent variations in EV number and size, several studies have examined variations in the molecular composition of EVs (Fig. 2B). To better understand the contribution of EVs-containing transporters in maintaining fluid and electrolyte balance and, in turn, blood pressure, Castagna et al. analyzed the expression of the renal thiazide-sensitive sodium chloride transporter (NCC) and prostasin in urinary exosomes purified from samples collected at various times during the day from healthy volunteers^104^. The results showed diurnal variation in NCC and prostasin concentration throughout the day, peaking at ~3–3:30 P.M., which paralleled the fluctuations of adiuretin and aquaporin, suggesting a role for circadian oscillation of EV content in fluid and electrolyte homeostasis^104^. Furthermore, these findings provide an optimal time window for the chronotherapeutic delivery of diuretic drugs targeting NCC for inhibition and, thus, for blood pressure control^104^.

miRNAs are also major constituents of EVs, and their selective enrichment in EVs is highly regulated. In a study aimed at understanding the contribution of night shift work on metabolic deregulation, Khalyfa et al. investigated the miRNA content of exosomes purified from blood samples collected at various times over a 24-h period from fourteen patients subjected to circadian misalignment following a simulated night-shift schedule for three days^105^. Microarray analyses of exosomal samples identified 62 miRNAs differentially expressed between day and night shift samples, with 10 reaching statistical significance^105^. Interestingly, treatment of naïve adipocytes with EVs from the simulated night-shift condition altered BMAL1 rhythmicity and led to the identification of significantly expressed peripheral target genes prominently involved in cell cycle regulation, glycolysis, and fatty acid metabolism^105^. Other studies have characterized diurnal variations of circulating cell-free miRNAs in plasma samples, not from purified EVs or bound to circulating proteins^106^, from healthy individuals exposed to ordinary daylight conditions^107^. The study reported that 26 of the 79 detected circulating miRNAs were rhythmic, based on cosinor-rhythmometry analysis, with many predicted to target clock-controlled and/or core clock genes^107^.

Most recently, Yeung et al. analyzed the proteome of small EVs isolated from circadian-synchronized primary mouse tendon fibroblast cells over a 48-h period. They identified reliably rhythmic proteins using label-free quantitative LC-MS-based proteomics combined with MetaCyle and Gaussian process analyses^108^. Functional enrichment analysis of EV proteins exhibiting circadian rhythmicity revealed that different pools of proteins peaked at distinct times. RNA-binding and ribosomal proteins peak earlier (6 to 10 A.M.) compared to cytoskeletal and extracellular matrix proteins, which peak later (10 to 11 P.M.)^108^. This is the first highly controlled in vitro study examining the role of the circadian clock in EV release and composition, where circadian synchronization was achieved and serum-free media was used to eliminate any EV contamination from the serum^108^. Of note, this study found no circadian variation in EV size, in contrast to previous in vivo studies^108^.

In summary, substantial evidence now supports the role of circadian rhythms in regulating diurnal variations in the release, size, and molecular composition of EVs (Fig. 2B). Studies demonstrate that these variations can influence biological processes such as coagulation, electrolyte balance, and metabolic regulation, with potential implications for disease biomarkers and therapeutic timing. Despite some inconsistencies in EV size data between in vivo and in vitro studies, these findings highlight the importance of understanding circadian influences on EV dynamics for improved biomarker analysis and chronotherapeutic applications.

### Rhythmic expression of genes involved in extracellular vesicles pathways

The results of the in vivo and in vitro studies discussed above support the potential role of the circadian clock in regulating EVs release and composition, with in silico tools helping to identify circadian-controlled genes involved in EV biogenesis.

We began by compiling databases, such as CircaDB, which offer a collection of curated circadian gene expression datasets from various tissues and species and can be queried for information on the expression of genes involved in EV biogenesis, cargo trafficking, and release that are potentially rhythmic and, thus, candidates for experimental studies^109,110^. CircaDB utilizes three separate algorithms for analyzing rhythmicity in transcriptome data: JTK_CYCLE^111^, Lomb-Scargle, and De Lichtenburg, allowing users to inspect the differences among the algorithm’s results^109^. An additional resource is MetaCycle, an analysis tool that is useful for analyzing rhythmic patterns in large time-series studies and for identifying rhythms in new datasets^112^. A third resource available in circadian biology research is CYCLOPS (CYClic Ordering by Periodic Structure). CYCLOPS, which is an algorithm designed to infer circadian phase from time-ordered but unphased data, is a complement to CircaDB, serving a different purpose and function^113^. It can assign circadian phases to datasets where circadian information is missing (e.g., lacking explicit time labels) or not well-defined, predicting circadian rhythms based on biological data, such as the Genotype-Tissue Expression Project (GTEx).

Using these resources, our initial analysis of mouse transcriptomic data was limited to transcripts identified as rhythmic by JTK_CYCLE with a probability cutoff of *P* < 0.05. Similarly, for the human transcriptome data, we applied a probability cutoff of *P* < 0.05 and a relative amplitude (rA) > 0.1. These cutoff values were selected to ensure statistical rigor while maintaining sensitivity to biologically relevant rhythmic signals. A *P*-value of <0.05 is widely accepted as a threshold for statistical significance, helping to minimize the chance of false-positive results. The relative amplitude threshold (rA > 0.1) was applied to filter out low-amplitude oscillations, which may not reflect meaningful biological rhythms, thus focusing on genes with stronger, more robust circadian patterns. These parameters balance detecting genuine circadian signals with reducing noise from weaker, potentially non-relevant, oscillations (Table 1).

**Table 1:** Rhythmically expressed genes involved in EV biogenesis, composition, and release

Genes of interest were queried in CircaDB, and rhythmicity was assessed using the JTK_Cycle method^109^. Full descriptions of gene acronyms are provided in the main text. GTEx, https://www.genome.gov/Funded-Programs-Projects/Genotype-Tissue-Expression-Project.

Using CircaDB as a straightforward query of experimental data to identify transcripts involved in ESCRT-dependent and -independent mechanisms exhibiting circadian oscillation, several significant hits were identified (Table I). RNA-seq data revealed that the *STAM1* subunit transcript is rhythmically expressed in various mouse tissues, including the heart, liver, and colon as well as in human tibial artery tissue (Table 1 and Fig. 2C). Given the role of the ESCRT-0 complex in endosomal trafficking and cargo sorting, the rhythmic expression of *STAM1* may influence the selective enrichment of proteins in exosomes.

Several mRNAs encoding subunits of the ESCRT-I complex, including *VPS28*, *TSG101*, and *UBAP1*, are rhythmically expressed in mouse tissues, such as the hypothalamus, liver, and lung, as well as in human visceral fat and in the coronary artery (Table 1 and Fig. 2C). As mentioned, TSG101 and UBAP1 play key roles in intercomplex interactions within the ESCRT machinery and the sorting of ubiquitinated cargo. For instance, downregulation of the *TSG101* homolog in yeast (*VPS23*) results in less efficient sorting of ubiquitinated cargo^114^. Therefore, the circadian regulation of *TSG101* and *UBAP1* transcription may impact cargo sorting into EVs. To date, no ESCRT-II components have been identified as being rhythmically expressed, and our analysis supports these findings. In contrast to ESCRT-II, genes encoding most subunits of the ESCRT-III complex are rhythmically expressed in both mouse and human tissues. These include chromatin-modifying protein/charged multivesicular body protein (CHMP) genes (*CHMP 1A/B*, *2A/B*, *4B/C*, *5*, and *7*), as well as one of the two paralogs of the VPS4 ATPase, *VPS4B* (Table 1 and Fig. 2C).

In conclusion, the circadian clock’s regulation of the ESCRT pathway provides a compelling mechanism for time-of-day control over processes like MVB biogenesis and cargo sorting. By precisely coordinating protein recycling and degradation, the clock optimizes cellular functions in line with daily rhythms. This temporal synchronization is crucial for maintaining cellular homeostasis and has significant implications for disease.

### Circadian regulation of multivesicular body trafficking routes

Trafficking of MVBs to the plasma membrane and exosome release into the extracellular space present additional steps in which the circadian clock may play a role. There are two primary fates for newly formed MVBs: degradation *via* lysosome/autophagosome or docking and fusion to the plasma membrane followed by secretion^65^. The trafficking of degradative and secretory MVBs is highly dependent on their interaction with the microtubule/actin cytoskeleton, motor proteins, and small Rab GTPases. Degradative MVBs are trafficked to the lysosome/autophagosome via dynein-dependent retrograde transport, while secretory MVBs are trafficked to the plasma membrane via kinesin-dependent anterograde transport. In these processes, RAB7, a member of the small Rab GTPase family that regulates key aspects of vesicle trafficking, including vesicle budding, cargo selection, transport, and fusion, plays a dual role in recruiting MVBs to lysosomes and facilitating exosome release. Rab GTPases facilitate these processes by recruiting various effector molecules (e.g., motors, kinases, phosphatases) to the vesicle membrane^115^. Importantly, Rab GTPases also serve membrane- and cell-type-specific functions. Several Rab GTPases implicated in MVB trafficking and EV release, including *RAB2B*, *RAB5A, RAB5C, RAB7*, *RAB9A, RAB11A, RAB14, RAB22A, RAB35*, and *RAB27A/B*, have been identified as rhythmic in mouse and human datasets (Table 1 and Fig. 2C). In addition to trafficking, Rab GTPases facilitate vesicle fusion via interaction with a superfamily of eukaryotic proteins, soluble N-ethylmaleimide-sensitive factor attachment receptors (SNARE), that are responsible for most endo and exocytic fusion events in the secretory pathway^116^. Several SNAREs, such as *VAMP7*, *SNAP23*, and *YKT6*, are also rhythmically expressed in mouse tissues (Table 1 and Fig. 2C). These findings strengthen the notion of a circadian-controlled temporal separation between EV generation and release, supporting existing experimental evidence of diurnal variations in EV release. Further research is essential to determine whether the circadian clock exerts membrane-specific or cell-type-specific control over EV release, potentially opening new avenues for understanding the timing and regulation of cellular communication processes.

### Circadian regulation of ESCRT-independent pathways

In addition to the classical ESCRT-dependent process, ESCRT-independent pathways of MVB biogenesis and MVB-independent EV biogenesis (e.g., microvesicle budding) have been characterized, with a full discussion available elsewhere^85,117^. Several genes encoding proteins involved in these ESCRT- and MVB-independent processes have been found to be rhythmically expressed from the datasets analyzed. Flotillins (FLOT1, FLOT2), membrane scaffolding proteins commonly associated with lipid rafts, play roles in ESCRT-independent processes such as clathrin-independent endocytosis (CIE), endosomal trafficking, ILV formation, and cargo sorting^117,118^. Flotillins also mediate cargo trafficking between ESCRT-0 and −1, suggesting a role in ESCRT-dependent pathways^119^. FLOT1 shows rhythmic activity in multiple mouse tissues, including the lung, liver, and aorta, while FLOT2 is rhythmic only in the lung^120^. However, as FLOT1 and FLOT2 regulate each other expression, it is likely that both proteins exhibit rhythmic patterns (Table 1). The GTPase ARF1, which is involved in microvesicle budding from the plasma membrane, is rhythmically expressed in human colon and mouse liver. Arrestin domain-containing protein 1 (ARRDC1)-mediated microvesicle (ARMM) release, an ESCRT-independent pathway from the plasma membrane, involves *ARRDC*, which is rhythmically expressed in mouse colon (Table 1). TSG101, whose transcription is rhythmic, is also thought to play a role in ARMM release. Together, these findings suggest that circadian clock components may regulate multiple EV release pathways.

### Circadian influence on EV biology manifests atmultiple levels of gene expression

In the previous sections, we primarily interrogated the oscillatory nature of mature transcripts whose protein products control various aspects of EV biology. This analysis was carried out in the context of extensive tissue- and species-specific data for which time series exist. While thorough, focusing solely on mature transcript oscillation captures only one aspect of circadian regulation. Additional layers of regulation, such as post-transcriptional modifications, need to be integrated into the analysis. The binding of clock proteins and the recruitment of RNApol II to regulatory regions in the genome result from rhythmic changes in histone modifications, which drive chromatin remodeling. Accordingly, a comprehensive analysis of circadian genome-wide level regulation and transcriptional profiles provides an opportunity for an in-depth assessment of the role of core clock components in various aspects of gene expression and post-transcriptional processing that relate to MVB biogenesis and cargo sorting.

Because circadian regulation in genes involved in vesicles biogenesis, loading, and release may occur at multiple levels prior to RNA maturation, we analyzed oscillations in core component occupancy and RNA polymerase II (RNApol II) recruitment, nascent RNA synthesis, and mRNA expression using publicly available, experimentally validated datasets^109,110,121–123^.

Several rigorous studies have examined the time-dependent evolution of the transcriptional landscape across tissues from both nocturnal laboratory and diurnal wild animals^121–123^. Today, available data allows for comprehensive spatiotemporal studies of gene expression that expand tissue specificity to consider the influence of environmental variations. To illustrate this potential gap and bring it to the attention of the reader, we provide three distinct examples for consideration. In the sections below, using available time-series data (including ChiP-seq, Nascent-seq, and RNAseq information), we reconstructed the multi-level regulation exerted by the circadian clock on various aspects of EV biology.

**Fig. 3:**
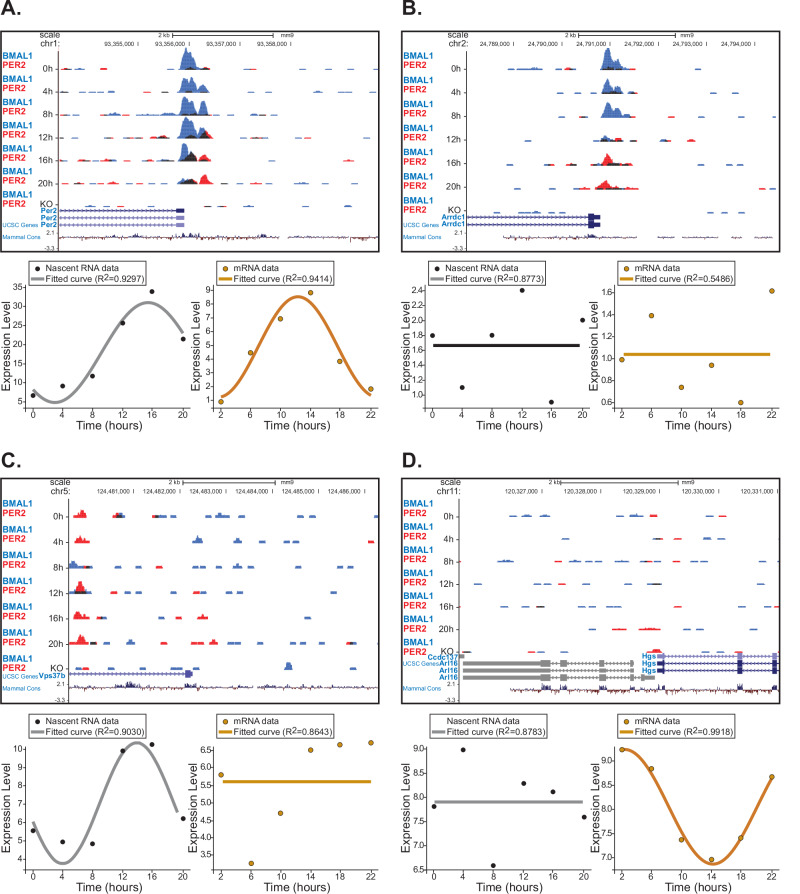
Circadian regulation of EV biogenesis occurs at multiple gene expression levels. ChIP-Seq, Nascent-Seq, and whole-transcriptome RNA-Seq analyses were conducted on mouse liver samples. Mice were entrained to 12:12 h dark/dark (ChIP-Seq) or light/dark (RNA-Seq) conditions prior to sample collection, which was performed every 4 h over a 24-h period. In all cases, the top panel displays the UCSC genome browser view of BMAL1 (blue) and PER2 (red) occupancy, overlaid, within ~2 kb of the promoter regions at the *Per2* (**A**), *Arrdc1* (**B**), *Vps37b* (**C**), and *Hgs* (**D**) loci. The mouse genome (NCBI37/mm9) was used as a reference. The ChIP-Seq dataset is from Koike et al. [Gene Expression Omnibus, accession number GSE39860^122^]. Data from knockout (KO) mice served as a negative control for each transcription factor. RNA-Seq data were previously published by Menet et al. Nascent RNA (bottom left panel, grey) and mRNA (bottom right panel, orange) abundance (reads per base pair) for each sample were used in the analysis [Gene Expression Omnibus, accession numbers GSE36872 and GSE36871, respectively^121^]. Analysis was conducted using the first replicate of samples collected every 4 h from 0 to 20 h (nascent RNA) and 4–22 h (mRNA). Data were fitted to a cosine function using the following equation: $y=B+A* \cos \left(\frac{2\pi * x}{T}-{phase}\right)$, where B represents the mesor of the rhythm, A is the amplitude, T is the period of the cycle, and phase indicates the phase shift. Curve fitting was performed using the curve_fit function from the SciPy optimize module to estimate the parameters.

Initially, we mapped the cistrome of *Arrdc1*, *Vps37b*, and *Hgs* to identify the binding of core clock components using available genome-wide chromatin immunoprecipitation sequencing (ChIP-seq) and RNAseq datasets collected at various circadian times from the liver of animals maintained in constant darkness^122^. Unlike Koike et al., high-throughput sequencing of nascent RNA (Nascent-Seq) and mRNA-seq datasets were from the liver of animals housed in a 12 h LD condition^121^. We chose these three genes for a proof-of-concept analysis because they represent different scenarios: the presence or absence of mature transcript oscillations, as identified in our CircaDB analysis (Table 1 and Fig. 3). For example, *Arrdc1* shows modest oscillation at the mRNA level in the colon, but not in the liver, *Vps37b* exhibits no rhythmicity at the messenger level, and *Hgs* shows a strong oscillation of the mature transcript in the liver (Table 1). Analysis of the well-characterized circadian mechanisms involved in *Per2* transcription using the same datasets are shown for reference (Fig. 3A). A heatmap illustrating the dynamics of activators (e.g., BMAL1) and repressors (e.g., PER2) binding within an ~2 kb regulatory region of cistromes reveals genomic elements where rhythmic binding occurs, highlighting sites that may play roles in circadian gene regulation. As shown in Fig. 3, time-dependent binding of BMAL1 and PER2 can be detected in *Arrdc1* and, to a lesser extent, in *Vps37b* (Fig. 3B, C, top panels). Maximum occupancy for BMAL1 occurs at canonical E-box and other regulatory elements at CT0 and CT8 for *Arrdc1* and *Vps37b*, respectively (Fig. 3B, C). However, while nascent and mature *Arrdc1* RNA levels remain constant, *Vps37b* exhibits a strong nascent RNA oscillation that does not translate into rhythmic mRNA expression (Fig. 3B, C, lower panels). Conversely, occupancy rhythms are not detected for either activators or repressors in the *Hgs* gene, and no oscillation is detectable in the nascent RNA despite strong rhythms of mature RNA expression (Fig. 3D).

These proof-of-concept analyses underscore the complexity of circadian gene regulation, where distinct regulatory layers, including chromatin remodeling, transcriptional initiation, and post-transcriptional modifications, can individually or collectively influence gene expression rhythms. The disparate rhythmic behaviors observed across *Arrdc1*, *Vps37b*, and *Hgs* highlight the need to consider each gene’s regulatory context when studying circadian influences, as reliance on mature transcript data alone may obscure significant regulatory dynamics that impact EV biology.

## Conclusions

Extracellular vesicles are essential for cell-to-cell communication as they transport a variety of cargo molecules crucial for maintaining physiological homeostasis. EVs also play significant roles in the initiation, maintenance, and progression of disease, including infectious, cardiovascular, and proliferative disorders. In this context, EVs, particularly exosomes, serve both as therapeutic targets and as biomarkers.

While the circadian clock regulates many biological processes, its influence in EV biology remains underexplored. To address this knowledge gap, multiple integrated approaches incorporating time-resolved sampling, multi-omics analyses, and diverse biological models will be essential. Diurnal variations in EV size, number, and composition have been observed in both healthy and diseased subjects, indicating circadian regulation of EV release and content. Understanding these temporal variations is essential for accurately interpretating EV-based biomarkers in diagnostic and therapeutic contexts, as time-of-day effects can impact measurements. However, the effects of circadian disruption on EV function and their role in disease progression are still largely unknown. As EVs continue to gain traction as clinically relevant biomarkers, standardizing collection and analysis protocols, including consideration of circadian timing, as emphasized by the MISEV 2023 guidelines, will be critical for their reliable application in both research and clinical settings.

## Data Availability

No datasets were generated during the current study. Datasets analyzed were GSE39860, GSE 36871, and GSE 36872 (Fig. 3).
